# Impalement injury by glass shard with delayed colonic perforation

**DOI:** 10.11604/pamj.2015.21.330.7676

**Published:** 2015-08-31

**Authors:** Adriá Rosat, Juan Manuel Sánchez, Cristina Chocarro, Manuel Barrera

**Affiliations:** 1 Department of General Surgery, Hospital Universitario Nuestra Señora de Candelaria, Ctra Del Rosario 145, 38010 Sta Cruz de Tenerife, Spain; 2 Transplantation Surgery Unit and General Surgery Service, Hospital Universitario Nuestra Señora de Candelaria, Ctra. Del Rosario 145, 38010 Sta. Cruz de Tenerife, Spain

**Keywords:** Impalement, glass, delayed colonic perforation

## Abstract

A 66-year-old man experienced a traumatic injury after a fall on top of a glass tea table, which caused some superficial lacerations all around the body. He was examined in the emergency room by a physician. The physician could not feel any foreign body upon wound exploration and sutured the laceration. Fourteen months after the injury, he developed progressive abdominal pain. On emergency room and abdominal x-ray showed a foreign body, which a CT scan revealed as an intraabdominal glass shard. The glass presumably impaled his abdominal wall as a result of his previous traumatic injury. The patient underwent laparotomy, which revealed a large glass (16x1cm) perforating the transverse colon. It was extracted and the perforation closed with a lineal stapler. There was no need of bowel resection and the patient was discharged home nine days after the intervention.

## Introduction

Most foreign bodies in the colon have been described as ingested orally or inserted rectally. Impalement injuries are those injuries produced from elongated objects that penetrate and remain imbedded in the human body [1]. We report a colonic perforation by foreign body in which the patient had a delayed presentation. Fourteen months after the initial injury, the patient presented as a consultation in the outpatient setting with left abdominal pain.

## Patient and observation

**Figure 1:**
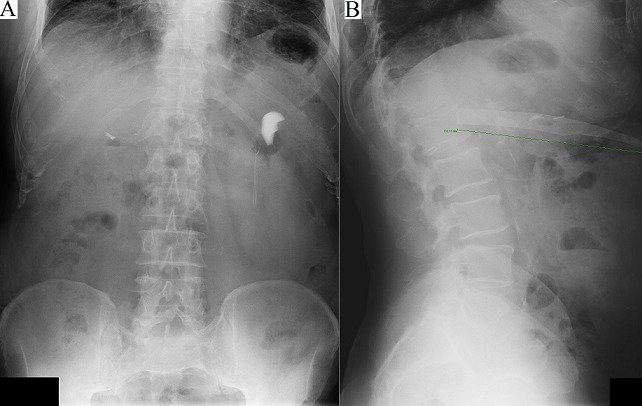
(A) supine abdominal x-ray, January 2014; (B) lateral abdominal x-ray, April 2015

A 66-year-old man was admitted in our outside hospital's emergency room due to abdominal pain. He reported that in January 2014 he fell on a glass tea table, which shattered, and he landed on the broken glass. His injuries were some superficial lacerations all around the body. An abdominal x-ray was taken in supine position (Figure 1 A). He was examined by a physician, who could not feel any foreign body upon wound exploration and sutured the laceration.

**Figure 2:**
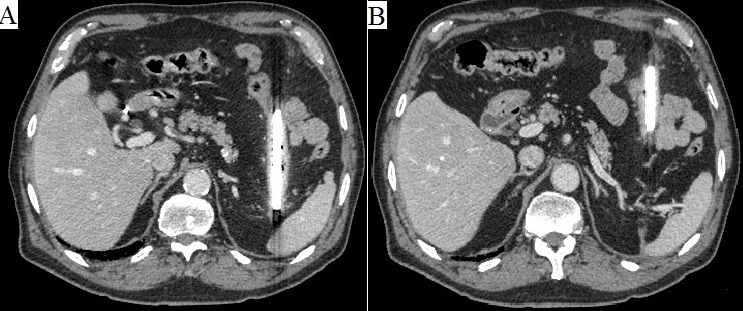
(A) abdominal CT scan: posterior end of the shard next to the spleen; (B) arrow points at the retroperitoneum scar, where the glass shard impaled the patient

Fourteen months later, he developed progressive pain on left abdominal wall. The physical examination showed an old scar on the left back and a protrusion on the left hypocondrium. Blood tests were unremarkable. A lateral abdominal x-ray revealed an image that made us suspect of a possible glass shard as cause of the pain (Figure 1 B). A CT scan was made and a very large glass shard was found inside the colon, just next to the pancreas, the left kidney and the spleen (Figure 2). Surprisingly, due to the location of the glass shard in the transverse colon, the patient did not have any obstructive symptoms probably further delaying his presentation. Preoperative broad-spectrum antibiotics along with tetanus immunoglobulin and tetanus toxoid were administered.

**Figure 3:**
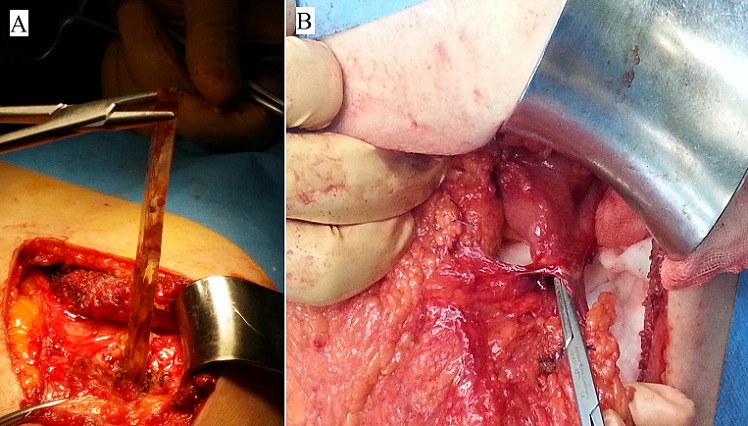
(A) glass shard extraction; (B) perforation on the antimesenteric edge of the transverse colon

The patient was taken to the operating room for a laparotomy to remove the foreign body. A left subcostal incision was made. Beneath the muscle we just felt the glass, and extracted it with no difficulty under visual control of the colon (Figure 3 A). No signs of peritonitis were seen. The perforation was on the antimesenteric edge of the colon (Figure 3 B) and we closed it with a lineal stapler. The postoperative course was uneventful and he was discharged home on the ninth postoperative day.

## Discussion

Impalement injuries usually are a result of accidental falls or collisions, homicidal attacks, or sexually perverted acts. Based on the mechanism of injury, impalement injuries can be classified clinically into three types. Type I impalement injury results from the impact of the body against an immobile object. Fall from height on to a stationary object is a typical example [2, 3]. Type II impalement injuries are due to the impact of a mobile object into the stationary patient. Homicidal attacks or sexually perverted acts may result in this type of injury. Type III impalement injury combines the mechanism of injury in type I and II, and results are due to an impact between a mobile patient and a mobile object.

There are certain broad recommendations for managing impalement injuries. If possible, the impaled object should not be manipulated or removed. This could release the tamponade effect of the object, resulting in massive bleeding [1, 4]. It can also result in splintering or breakage of the object [5]. All impaling objects are to be considered as highly contaminated, thus, broadspectrum antibiotic coverage and tetanus prophylaxis should be administered at the time of presentation in the hospital [1, 5].

The incision must be made in a manner that will provide adequate exposure for vascular control and complete visualization along the path taken by the penetrating object. The incision should also facilitate removal of the object under direct vision. Hence, unconventional or non-standard incisions may sometimes be required [1, 5]. The impaled object should be removed only after complete exploration and proper vascular control [1].

Intestinal perforations by foreign bodies may be acute or chronic. Acute perforation, which is most likely caused by a pointed foreign body, tends to occur in the small intestine. Chronic perforation is most likely caused by a nonpointed foreign body and tends to occur in the sigmoid colon [6]. Acute perforations are usually clinically remarkable, and the history is short as patients present with acute peritonitis. The foreign body is probably pushed through the wall by peristaltic activity. At operation, no adhesions are found walling off the inflammatory process [6]. In contrast, patients with chronic perforation (such as ours) may present with nonspecific complaints of subacute or chronic nature. The presumed mechanism is ulceration caused by pressure necrosis; inflammation may be limited by the formation of adhesions, abscesses, or inflammatory masses. This mechanism seems to be supported by the multiple reports of prolonged time between ingestion and perforation [7, 8].

We still wonder why the patient did not have any abdominal symptoms when he first got the colon perforated. Although glass can be seen on a radiograph and allow diagnosis and proper treatment, it is still difficult, and this is not the first time in which a glass shard is dismissed as artifact or remains unseen on an x-ray until it causes different symptoms [9].

This case has 3 unique features. First, the mechanism in which the injury occurred makes this an extraordinary case. After an impalement injury, the patient had a colonic perforation with a delayed presentation. Fourteen months after the initial injury, he presented with atypical left abdominal pain. Second, the shard of glass impaled the colon just between the pancreas, the left kidney and the splee, leaving them undamaged. Third, this unique impalement injury was ultimately treated with no need of bowel resection. There is only one similar case reported in the literature, which needed bowel resection [10].

## Conclusion

After establishing a detailed history from the patient, the key step for the diagnostic in our case was an abdominal x-ray in lateral projection. Injuries caused by glass can happen not only by accident but also by aggression, leaving small shards inside the patient. This case tries to remember the huge help that a simple x-ray image can give us.
